# The use of DIY (Do it yourself) sampling and telemonitoring model for COVID-19 qPCR testing scale up

**DOI:** 10.1371/journal.pone.0259398

**Published:** 2021-11-04

**Authors:** Olufemi Samuel Amoo, Funmi Adewara, Bosun Tijani, Tochukwu Ifeanyi Onuigbo, Amaka Stephanie Ikemefuna, Joy Isioma Oraegbu, Tazeen Rizvi, Azuka Okwuraiwe, Chika Onwuamah, Joseph Shaibu, Ayorinde James, Greg Ohihoin, Fehintola Ige, Dorcas Kareithi, Agatha David, Steven Karera, Hammed Agboola, Anthony Adeniyi, Josephine Obi, Dominic Achanya, Ebenezer Odewale, Osaga Oforomeh, Gideon Liboro, Olayemi Nwogbe, Oliver Ezechi, Richard Adegbola, Rosemary Audu, Babatunde Salako

**Affiliations:** 1 Department of Microbiology, Nigerian Institute of Medical Research, Yaba, Lagos, Nigeria; 2 Mobihealth International Limited, Abuja, Nigeria; 3 Co-creation Hub, Lagos, Nigeria; 4 Department of Biochemistry, Nigerian Institute of Medical Research, Yaba, Lagos, Nigeria; 5 Clinical Science Department, Nigerian Institute of Medical Research, Yaba, Lagos, Nigeria; 6 Department of Library and Information Technology, Nigerian Institute of Medical Research, Yaba, Lagos, Nigeria; Konkuk University, REPUBLIC OF KOREA

## Abstract

The first case of COVID-19 in Nigeria was recorded on February 27, 2020, being an imported case by an Italian expatriate, to the country. Since then, there has been steady increase in the number of cases. However, the number of cases in Nigeria is low in comparison to cases reported by other countries with similar large populations, despite the poor health system prevailing in the country. This has been mainly attributed to the low testing capacity in Nigeria among other factors. Therefore, there is a need for innovative ways to increase the number of persons testing for COVID-19. The aim of the study was to pilot a nasopharyngeal swab self-sample collection model that would help increase COVID-19 testing while ensuring minimal person-to-person contact being experienced at the testing center. 216 participants took part in this study which was carried out at the Nigerian Institute of Medical Research between June and July 2020. Amongst the 216 participants, 174 tested negatives for both self-collected samples and samples collected by Professionals, 30 tested positive for both arms, with discrepancies occurring in 6 samples where the self-collected samples were positive while the ones collected by the professionals were negative. The same occurred in another set of 6 samples with the self-collected samples being negative and the professional—collected sample coming out positive, with a sensitivity of 83.3% and a specificity of 96.7%. The results of the interrater analysis are Kappa = 0.800 (95% CI, 0.690 to 0.910) which implies an outstanding agreement between the two COVID-19 sampling methods. Furthermore, since p< 0.001 Kappa (k) coefficient is statistically different from zero, our findings have shown that self-collected samples can be reliable in the diagnosis of COVID-19.

## Introduction

Human coronavirus belongs to a group of viruses that causes an infection in humans with four main sub-groupings known as alpha, beta, gamma, and delta coronavirus [1]. Before the advent of SARS-CoV-2, other types of coronaviruses including, alpha coronavirus (229E, NL63); beta coronavirus (OC43, HKU1, MERS-CoV-causing Middle East Respiratory Syndrome, or MERS), SARS-CoV-causing severe acute respiratory syndrome, or SARS, have been described [2–4].

SARS-CoV-2 (the novel coronavirus that causes coronavirus disease 2019, or COVID-19); the latest addition to the coronavirus family [5] was first identified on 17th of November 2019 from a 55-year-old Hubeian citizen but officially announced by the Chinese Government on 8th of December 2019 [6]. By January 2020, the disease was named COVID-19 and declared as an epidemic by the World Health Organisation (WHO) due to the unprecedented nature of the disease outbreak and later declared a pandemic on March 11, 2020 [7].

From the outset of the coronavirus outbreak in China, there has been an exponential increase in infections spreading across 212 countries and territories. Statistics have shown that as of the 4^th^ of September 2021, recorded cases of coronavirus had surpassed 221 million with over 4.5 million deaths worldwide. It was reported that many countries battled with challenges of diagnosis and effective treatment for the diseases [8].

The Nigerian Centre for Diseases Control (NCDC) rolled out eligibility for COVID-19 testing in the country; (1) Any person showing symptoms including fever, cough, or difficulty in breathing. (2) History of travel to and more than 24 hours of transit through any high-risk country with widespread community transmission of SARS-CoV-2. (3) Close contact with a confirmed case of COVID-19 (4). Exposure to a healthcare facility where COVID-19 case(s) have been reported [9]. The first case in Nigeria was recorded on February 27, 2020, being an imported case of an Italian expatriate and the number of cases keeps rising, affecting about 35 states so far with reported cases over 130,000 as of January 31, 2021 [10]. So far, only about 1 million of the Nigerian population have been tested for COVID-19. Hence the need for an effective means to identify and test for cases among the teeming population of about 200 million people.

Though measures are being put in place in ensuring that testing centers are made available to meet the growing need for people to be tested in Nigeria, there is still a massive gap in the testing capacity as infections soar. To expand testing in the country, there is a need for innovative ideas that will reduce or eliminate long waiting hours at testing sites, control crowds which is an avenue for effective spread of the virus, as well as minimize person-to-person contact during testing. A self-service diagnostic testing model that would expand testing was proposed elsewhere [11] but it has not been tried and evaluated in Nigeria. The COVID-19 DIY sampling testing model would minimize person to person contact as well as reduce crowds presenting at testing centers. This study aims to evaluate the efficiency and success of home testing by employing a self-sampling model thereby reducing the burden on health workers and the spread of COVID-19 infection.

## Methods

### Patients recruitment

The drive-through COVID-19 test Centre located at the Nigerian Institute of Medical Research (NIMR), is one of the largest testing centers in Lagos, with an average of 120–140 people presenting for testing daily during surge periods. Individuals who participated in this study were selected from people presenting for testing at the drive-through COVID-19 test Centre. The Study procedure was explained to each individual presenting for sample collection, only those that gave verbal consent were enrolled in the study. A total of 216 people consented to take part in the study which took place between June and July 2020. Participants presenting, who were below 15 years of age and who too weak to carryout sample collection by themselves, were excluded from the study.

Participants who gave their consent after being briefed about the study, were asked to watch a video noting the procedure, after which they were given swabs and transport medium to collect their samples individually. Participants were monitored both on site by health professionals and remotely using ‘Mobihealth consult’, a telemonitoring platform developed by Mobihealth international. This application allows health professionals to remotely monitor the sample collection process, guiding each participant to enable them collect their samples properly. About 20 mins after, their samples were also collected by professionals (healthcare workers) on-site which is the gold standard for sample collection. All samples were taken to the laboratory and tested within 24 hours of collection using Roche COBAS 6800. The COBAS 6800, has a Positive Percent Agreement of 100% and a Negative Percent Agreement of 100%. With a Sensitivity and Specificity at 95% Confidence interval of (95% CI: 86.7% - 100%) and (95% CI: 96.3% - 100%) respectively.

### Statistical analysis

Descriptive statistics were computed by finding mean and standard deviation for continuous (numerical) variables and frequencies for categorical variables. Sensitivity and specificity analysis of the DIY test was done to determine if the test is sensitive for detecting the disease (it is positive for most people having the disease) and if it is specific (it is positive for a small percentage of those without the disease). This was calculated using:

Sensitivity=[(TruePositive)/(TruePositive+FalseNegative)]*100

Specificity=[(TrueNegative)/(TrueNegative+FalsePositive)]*100

The interrater reliability analysis (Cohen’s Kappa statistics) was also used to measure the level of agreement between the result of the samples collected by professionals and the samples collected by individuals. This was calculated using the formula

$$\kappa =\frac{p_{o}-p_{e}}{1-p_{e}}=1-\frac{1-p_{o}}{1-p_{e}}$$

where:

Po = the relative observed agreement among raters.
Pe = the hypothetical probability of chance agreement.

### Ethical approval

Ethical approval was obtained from the Nigerian Institute of Medical Research Institutional Review Board (IRB/20/044).

## Results

### Demographic characteristics

**Fig 1:**
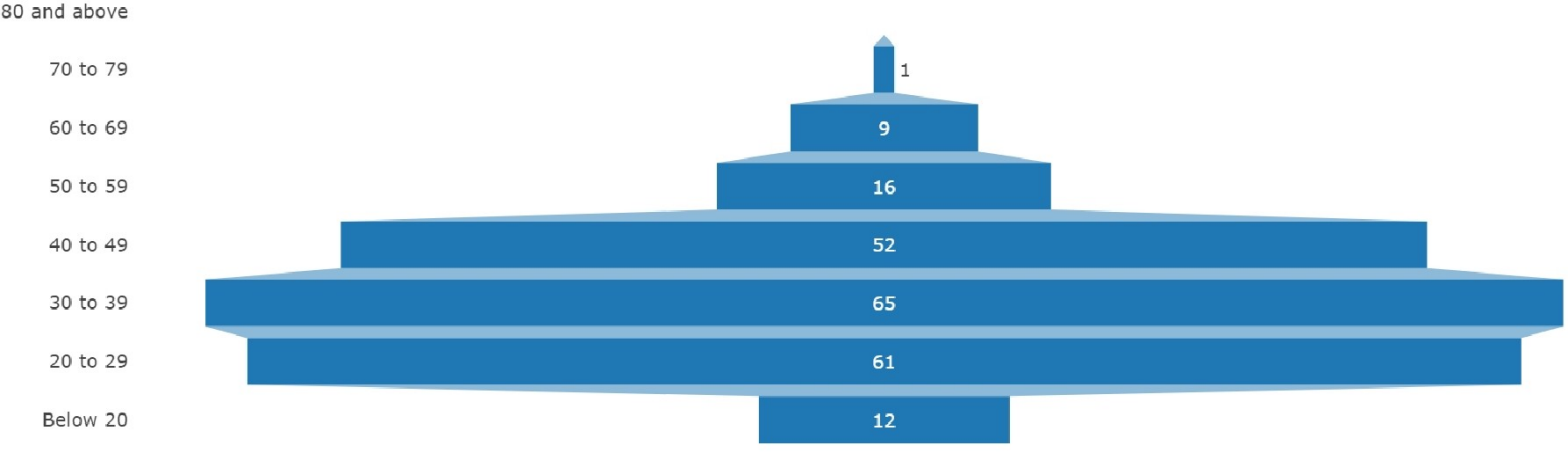
Respondent’s age distribution.

A sample of 216 participants was used to assess the efficiency and success of self-testing. The distribution of the participants by age is shown in Fig 1.

The mean age of study participants was 35 (±14.2) years old and among all the age groups, the majority (30%) were 30–39 years old.

In addition, more than half (56.5%) of the participants were male. The most frequent marital status was married status (58.3%) followed by single (39.8%), and then separated (1.4%) while divorced status was less represented (only 1 person was divorced).

### Exposure to COVID-19

**Fig 2:**
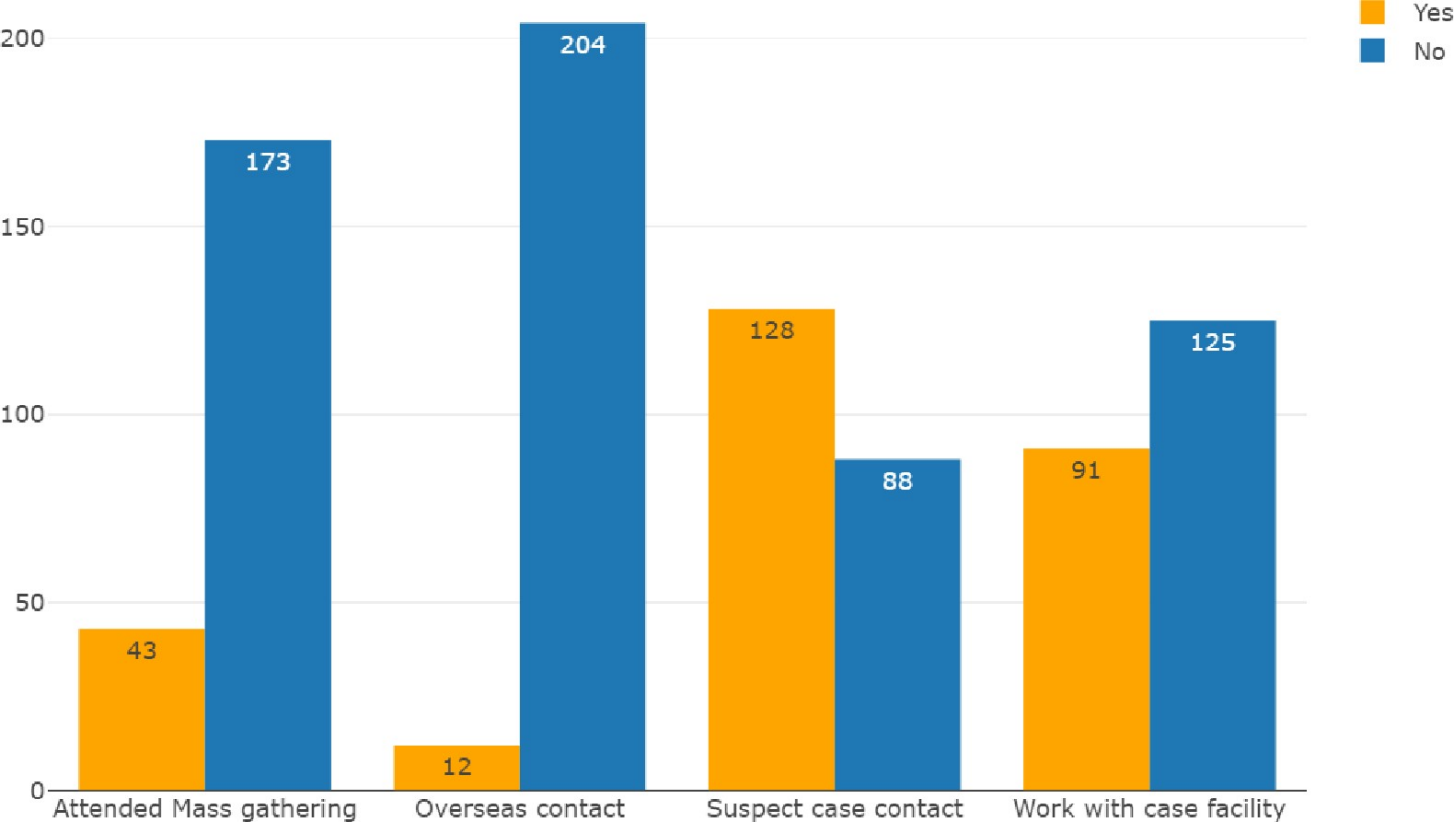
Respondents exposure.

Out of the 216 participants, 128 (59.3%) had contact with suspected cases, 91 (42.1%) participants were employed in a care facility, while 43(19.9%) had attended mass gathering and 12 (5.6%) had contact with overseas travelers (Fig 2).

### Symptoms and pre-existing conditions

Even though many of the study participants n = 195 (90.3%), had no pre-existing conditions, among those with pre-existing conditions n = 21(9.7%), chronic liver disease and heart disease were the most reported conditions. Furthermore, 52.3% of study participants had developed at least a COVID-19 symptom with fever (32.4%), sore throat (24.5%), and dry cough (23.6%) being the most developed symptoms. In addition, one person had previously tested COVID-19 positive (Table 1).

**Table 1:** COVID-19 symptoms and pre-existing health conditions.

Indicator	Category	Base	Number	Percentage
Symptoms	No symptoms	216	103	47.7%
	Fever	216	70	32.4%
	Sore throat	216	53	24.5%
	Dry cough	216	51	23.6%
	Nasal congestion	216	38	17.6%
	Shortness of breadth	216	37	17.1%
	Running nose	216	31	14.4%
	Diarrhea	216	28	13.0%
	Abdominal pain	216	28	13.0%
	Headache	216	5	2.3%
	Loss of smell	216	5	2.3%
	Fatigue	216	4	1.9%
	Chest pain	216	4	1.9%
	Muscle pain	216	3	1.4%
	High blood pressure	216	3	1.4%
	Dark urine	216	2	0.9%
	Indigestion	216	1	0.5%
	Insomnia and sweating	216	1	0.5%
	Loss of taste	216	1	0.5%
	Back pain	216	1	0.5%
	Previously tested positive	216	1	0.5%
Pre-existing conditions	Asthma	21	1	4.8%
	Chronic liver disease	21	9	42.9%
	Chronic cough	21	2	9.5%
	Diabetes	21	2	9.5%
	Heart disease	21	7	33.3%
	Hemorrhoid issue	21	3	14.3%
	Ulcers	21	3	14.3%
	Obesity	21	4	19.0%
	Pregnancy	21	3	14.3%
	Chronic lung disease	21	1	4.8%

### Sensitivity and specificity test

The table below presents the test results of samples collected by participants (DIY) in comparison with results of samples collected by healthcare workers. There are 30 true positive tests compared to 6 false-negative tests. The sensitivity is 100(30)/(30 + 6) = 83.3%. This means that 83.3% of the individuals with the COVID-19 will have a positive screening test when using the DIY test. The 6 false-positive tests and 174 true negative tests give specificity of 100(174)/(174 + 6) = 96.7%. This means that 96.7% of the individuals without the COVID-19 will test negative when they use the DIY test (Table 2).

**Table 2:** Testing results of DIY diagnosis tool and LAB collection.

DIY COLLECTION	HEALTHCARE WORKERS COLLECTIONS	
	Tested positive	Tested negative
Tested positive	30	6
Tested negative	6	174

Interrater reliability test: The results of the interrater analysis are Kappa = 0.800 (95% CI, 0.690 to 0.910) which implies an outstanding agreement between the two COVID-19 sampling methods.

## Discussion

The need for an increase in testing capacity and to reduce contact between health workers and individuals suspected of COVID-19 as well as to compare the efficiency of self-collected samples with samples collected by professionals (healthcare workers) describe the aims of this study. Before there can be a roll-out of self-sampling collection kits, there is a need to ascertain and make sure that samples collected by untrained individuals would produce the comparable results as those collected by professionals. The gold standard sampling technique for the confirmation of COVID-19 is the nasopharyngeal and oropharyngeal sampling [12].

Male participants represented more than half of the study population. This corresponds with statistics from the two hardest-hit European countries which showed that the male gender accounted for the majority of COVID-19 deaths in Italy and twice as many men as women in Spain [13]. Recent studies have shown that the male population was the most susceptible gender to being infected with COVID- 19 than females [14]. This trend is attributed to genetic, immunologic, and behavioral patterns exhibited by the male and female gender [15].

Behavioral lifestyle patterns among men and women tend to implicate COVID-19. The male gender has shown to have an increased lifestyle pattern of drinking and smoking than women [16]. The female gender tends to show higher compliance with preventive measures such as regular handwashing, use of alcohol-based sanitizers, face mask, and maintaining social distancing [17].

Furthermore, during this study, different age groups were seen among the participants. Previous studies have shown that the most vulnerable age group comprising 60 years and above are made up of the elderly and immunocompromised patients [18]. However, most of the participants in this study were found among the age group 30–39 years and the mean age 35 years. This may not be unrelated to the relatively young population structure of Nigeria, indicating that individuals of ages ≥ 65 years as a share of total population was 2.7% in 2020 [19]. These findings correlated with studies from CDC, 2020 which attributed the cause to occupational and behavioral factors thus exposing this age group to a higher risk of infection [20] Also, these younger adults tend to not adhere maximally to non-pharmaceutical prevention strategies for SARS-CoV-2 infection such as social distancing and avoidance of crowded social gatherings. They also tend to be more asymptomatic or have mild symptoms thereby transmitting this infection to the most vulnerable age groups made up of the elderly and immunocompromised.

The marital status of the participants was also considered, among all participants, married people were discovered to have the highest percentage among the participants. One of the preventive measures in managing the spread of COVID-19 includes practicing social distancing. However, this method seems to be difficult among household members which might be unknowingly infected with COVID-19. Reports show that among people living in the same house, the rate of infection is higher among adults, with an infection rate of 27.8% amongst spouses [21]. Thus, early detection and quarantining of infected patients can help limit COVID-19 spread between spouses and the household at large.

Half of the study participants were symptomatic, amongst which symptoms such as fever, cough, and sore throat were the most experienced. This correlated with data from previous reviews on COVID-19 studies in which the most common symptoms are the same [7]. Although many of the participants in this study did not have pre-existing conditions, the majority of the small percentage with pre-existing conditions had chronic kidney disease, this is in contrary to the findings of Richardson [22] which inferred that heart disease was a major pre-existing condition. Furthermore, a greater part of our study participants was in contact with a confirmed or suspected COVID-19 case and this form of exposure is said to be responsible for the high infectivity rate for COVID-19 [23].

Participants in this study were made to watch a video of how the procedure is done before trying it on site. This is to guide the participants in order to ensure a good enough sample collection procedure. Results from this study showed a specificity of 96.7% and a moderately high sensitivity of 83.3%, in comparison with the professionally collected samples. These findings differed from a study by Abdollahi *et al*. [24] with a sensitivity of less than 70% and a specificity of less than 95%, in which, it was concluded that samples collected by professionals were more reliable than self-collected samples. The study by Abdollahi, however, was carried out on a small sample size of 50 patients, which might be a factor in the result realized, also, the median age of participants was 56 years compared to our study which had a median age of 35 years. Another factor that could be responsible for such a contradiction is that 9 out of 11 patients who reported having underlying conditions according to Abdollahi, [24] have severe medical conditions. They concluded that self-collection is not recommended for patients with severe medical conditions. In our study, 47.7% of the participants were asymptomatic with the remaining showing mild to moderate symptoms, this could be another factor that contributed to the high sensitivity and specificity rate of self-collected samples.

In another study by seaman [25] a meta-analysis was carried out on fourteen papers that focused on the effectiveness of self-sample collection compared with professional-swabbing in the diagnosis of influenza in symptomatic individuals. The sampling technique required for influenza and that for SARS-COV-2 are similar with both requiring specimens using nasopharyngeal and oropharyngeal swabbing as well as a nasopharyngeal wash which is not so common in SARS-COV-2 detection. They found out that self-sampling was highly acceptable, simple, and comfortable, and that self-sample collection findings may be affected slightly by measurement errors. The pooled sensitivity of 87% and specificity of 99% were reported from nine studies. Hence reports from these studies were in concordance with our work which showed that self-sampling is a reliable method of sample collection for COVID-19.

Dennis McCulloch [26] in a study that recruited 185 participants who carried out self-swabbing at home, unsupervised showed a sensitivity of 80% and a specificity of 97. 9% which is similar to what has been obtained in our study. However, the majority (158) of their study participants were health care workers from drive-through clinics with fourteen of them eventually testing positive. Our study excluded health workers so as not to create a bias in sample collection based on technical know-how. The results of the interrater analysis which is Kappa = 0.800 (95% CI, 0.690 to 0.910) implies an outstanding agreement between the two COVID-19 sampling methods. Furthermore, since p< 0.001 Kappa (k) coefficient is statistically different from zero, our findings have shown that self-collected samples can be reliable in the diagnosis of COVID-19.

It is important to ascertain the reliability of samples collected by untrained individuals as the majority of those who would be involved would have little or no skill in self-sample collection. This is to make sure that samples collected individually would produce the same results as the ones collected by professionals.

## Conclusion

The introduction of self-sampling in SARS-COV-2 detection is to increase testing capacity, especially while the pandemic lingers, self-sampling can also be effective in surveillance which would enable larger portions of people to be reached within a shorter period. This study elucidates that self-sample collection can indeed be reliable and should be encouraged as a means to expand testing for COVID-19.

### Limitation of study

Poor internet connection limited the extensive use of telemonitoring system, as some participants had issues with connection, hence not being able be assessed by the health professionals who are off site. However, health professionals on site were available to carry out monitoring in such cases.

### Recommendation

We recommend that testing centers implement this strategy so as to reduce waiting time during sample collection and also reduce contact with healthcare professionals. This would help curb the transmission of this virus from patient to healthcare worker and enable a more rapid sample collection process. This sample collection method can be used at home or in offices, for people who are unable to make it to testing centers and need to have their samples collected. This can be achieved by simply sending the sample collection kits to them and return to the testing laboratory through a courier service.
